# Dermoscopic characteristics of membranous aplasia cutis congenita: Report of 56 cases

**DOI:** 10.1111/srt.13200

**Published:** 2022-08-21

**Authors:** Li Zhang, Zhao Zhang, Hong‐Jing Jiang, Yun‐Jing Pu, Hong Shu, Ya‐Dong Li

**Affiliations:** ^1^ Kunming Children's Hospital Kunming China; ^2^ Institute of Medical Biology Chinese Academy of Medical Sciences Kunming China

**Keywords:** congenital, dermoscopy, membranous aplasia cutis congenita

## Abstract

**Background:**

Membranous aplasia cutis congenita (MACC) presents at birth characterized by oval epidermis defect. Skin lesions with MACC have various clinic manifestations. In recent years, the usefulness of trichoscopy (scalp dermoscopy) has been reported for hair loss diseases. However, the dermoscopic features of MACC were mostly reported by case reports.

**Objectives:**

To summarized the obvious dermoscopic characteristics of MACC.

**Materials & Methods:**

These 56 cases met the clinical diagnostic criteria for MACC without forceps delivery complications or other birth injuries. To find the dermoscopic characteristics of MACC by summarizing 56 infants' dermoscopic pictures.

**Results:**

The dermoscopic manifestation of MACC are characterized by hair follicle openings and hair deficiency in the center of skin lesions, translucent epidermis, hair root and hair bulb arranged along the margins of skin lesion.

**Conclusion:**

The typical dermoscopic characteristics of MACC could help clinicians to early diagnose and differential diagnosis.

## 1 INTRODUCTION

Membranous aplasia cutis congenital (MACC) is a common clinical subtype of congenital ACC and indicates the membranous covered oval defect on the surface at birth. Clinical symptoms of MACC are manifested in blister, bulla, or cicatrix with the deficiency of hair in the center.^1^, ^2^, ^3^ Skin lesions with MACC have various manifestations with obvious dermoscopic characteristics, including hair follicle openings and hair deficiency in the center of skin lesions, translucent epidermis, and hair root and hair bulb arranged along the margins of skin lesion.^3^, ^4^, ^5^, ^6^ Dermoscopic manifestations of MACC in 56 cases were herein summarized to assist clinicians to conduct early and differential diagnosis.

## 2 REPORT OF 56 CASES

These 56 cases met the clinical diagnostic criteria for MACC without forceps delivery complications or other birth injuries. Among them, there were 21 (37.5%) male and 35 (62.5%) female cases. Besides, 33 (58.9%) cases had MACC on the parietal lobe of skull, four (7.1%) cases on the left lobe, three (5.4%) cases on the right lobe, and 16 (28.6%) cases on occiput. In addition, 53 (94.6%) cases developed a single lesion, and the other three (5.4%) cases suffered from multiple lesions. Three lesions were found on the most serious case. Besides, four (7.1%) cases mainly had blister, seven (12.5%) cases had erythema, and flat cicatrix was detected in 45 (80.4%) cases.

The combination of hair collar sign with MACC can serve as the relatively specific indicator of neutral tube defects, and it is prone to be associated with hydrocephalus, meningeal arteriovenous fistula, epilepsy, primary optic atrophy, and nevus flammeus.^7^, ^8^ Among our cases, 17 (30.4%) cases had hair collar sign with MACC. B‐mode ultrasound revealed that only two cases had intracranial traffic and meningocele, and computed tomography showed that one case had cerebral edema and leukomalacia. Capillary malformation around skin lesions has been found in 11 (19.6%) cases, and solid mixed echogenic masses were detected by B‐mode ultrasound in four (7.1%) cases, without blood flow signals.

Under dermoscopy, four (7.1%) cases were yellow in the background, 15 (26.8%) cases were flesh‐colored, 26 (46.4%) cases were red, and 11 (19.6%) cases appeared as combination of flesh‐colored with red. Among these 44 (78.6%) cases who were found with hair root and hair bulb along the margin of skin lesions, 21 (37.5%) cases had hair root and hair bulb arranged radially along skin lesions, 18 (32.1%) cases had linear‐or arc‐arranged lesions, and five (8.9%) cases had disorderly arranged lesions. A fine reticular and branched vascular network could be detected in 40 (71.4%) cases, seven (12.5%) cases were identified with spot‐or sphere‐shaped blood vessels, and nine (16.1%) cases were found with no obvious change in the structure of blood vessels. Moreover, 39 (69.6%) cases were detected with a pepper‐like focally‐distributed pigment structure, 22 (39.3%) cases were found with a degenerative structure, and eight (14.3%) cases had unobvious translucent epidermis along the margins. All (100%) cases had disappeared hair follicle openings and fingerprints, without hair growth (Table 1).

**TABLE 1:** Characteristics of 56 MACC cases under dermoscopy (%)

	Background				Distribution of hair root and hair bulb				Blood vessel			Translucent epidermis		Other structure	
Skin lesions	Yellow	Flesh‐colored	Red	Combination	Radial	Linear, arcshaped	Disorderly	Unobvious observation	Spot‐ or sphere‐shaped	Reticular	None	Obvious	Unobvious	Pigment	Degenerative structure
Blister	4	0	0	0	0	4	0	0	0	4	0	4	0	0	0
Erythema scale	0	0	7	0	1	3	2	1	4	3	0	5	2	6	2
Flat cicatrix	0	15	19	11	20	11	3	11	3	33	9	39	6	33	20
Total	4 (7.1)	15 (26.8)	26 (46.4)	11 (19.6)	21 (37.5)	18 (32.1)	5 (8.9)	12 (21.4)	7 (12.5)	40 (71.4)	9 (16.1)	48 (85.7)	8 (14.3)	39 (69.6)	22 (39.3)

**FIGURE 1:**
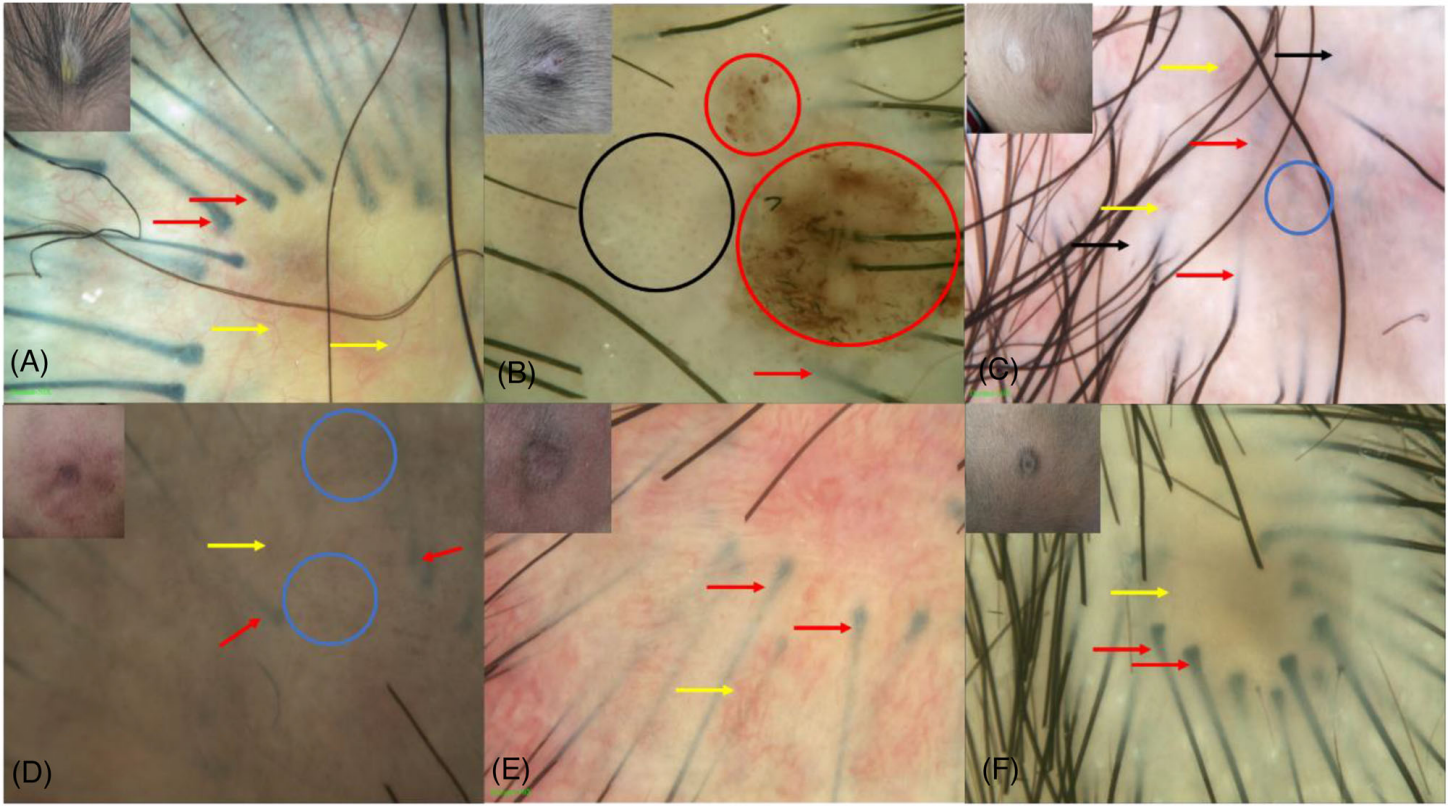
Dermoscopic features of the different clinical manifestations of MACC (A) blister type of MACC: yellow background, vascular network (the yellow arrow), translucent epidermis along the margins, arc‐arranged hair root, and hair bulb (the red arrow); (B) erythema type of MACC: dark red background, hair root along the margins (the red arrow), spot‐or sphere‐shaped blood vessels (the black circle), reddish brown scab and hemorrhage (the red circle); (C and D) flat cicatrix type of MACC: flesh‐colored and light dark background, pepper‐like hyperpigmentation (the blue circle), translucent epidermis and hair root and hair bulb arranged along the margins, (the red arrow) with degenerative structure (the black arrow); (E) combination of vascular malformation with MACC: red background, reticular and linear blood vessels (the yellow arrow), hair root, and hair bulb are arranged along the translucent epidermis in an arc shape (the red arrow); (F) combination of hair collar sign with MACC: hair root and bulb are arranged along the translucent epidermis in an arch shape (the red arrow), and vascular network (the yellow arrow).

Cases of blisters with serous fluid were characterized by a fine vascular network in the yellow background (Figure 1A). Cases of erythema mainly had focally distributed spot‐or sphere‐shaped blood vessels in the red background (Figure 1B). Cases of flat cicatrix typically had focally distributed pepper‐like hyperpigmentation in the flesh‐colored and light red background, with a degenerative structure in some parts (Figure 1C,D). As for cases with combined vascular malformation, their skin lesions were mainly manifested in reticular and linear blood vessels under dermoscopy in the red background (Figure 1E). In the case of hair collar sign around skin lesions, hair root and hair bulb could be visible along the margin of skin lesions arranged in an arc or radial pattern within the translucent epidermis (Figure 1F).

## 3 DISCUSSION

In recent years, the usefulness of trichoscopy (scalp dermoscopy) has been reported for hair loss diseases. The center of skin lesions of MACC mainly manifests in hair follicle openings and hair deficiency, and translucent epidermis is associated with the edge of skin lesions, where dermatrophy‐induced vascular network is visible, and hair root and hair bulb are arranged along the margins.^3^, ^4^, ^5^, ^6^ Due to the abnormal epidermal development, resulting in thinning of skin, MACC appeared translucent under dermoscopy. However, we found that translucent margins did not typically appear in erythema and cicatrix rashes, possibly because our observations were subjected to inflammatory response or the hypertrophic collagen of cicatrix in skin lesions. When skin is translucent, hair root and hair bulb are clearly observed in the cortex under dermoscopy. Some scholars described radial‐patterned hair root and hair bulb in MACC under trichoscopy as “hair bulbs arranged radially along hair‐bearing margins”^3^ and “golf club set”.^6^ Our findings revealed that hair root and hair bulb were mainly arranged in radial or linear and arc shapes. While in the late stage cicatrix, as some hair roots were not completely presented, they were disorderly arranged or observed in a less obvious manner. Rout et al.^9^ reported a case whose atrophic skins had white reticular stripes. These stripes cut skin lesions into cobblestone shapes. However, such manifestations were not found in our cases.

MACC often needs to be distinguished from nevus sebaceus and spotty pelada. For nevus sebaceus, yellow and white spots irrelevant to hair follicle openings are mainly found in skin lesions.^10^ Changes often happen in hair in the case of triangular temporal alopecia, spotty pelada, tinea capitis, and trichotillomania under dermoscopy, with unchanged fingerprints. These features can help distinguish MACC from other congenital or acquired hair loss as an important indicator. Triangular temporal alopecia typically manifests in dryer and finer fluffs distributed in skin lesions, compared with normal hair. Spotty pelada features yellow or black spots, and tinea capitis reveals comma‐shaped hair, whereas broken and flame‐shaped hair can be manifested in trichotillomania.^6^

## Data Availability

Data available on request from the authors.
